# Tuberculosis-Associated Hemophagocytic Syndrome Mimicking Malignant Lymphoma: A Case Report

**DOI:** 10.7759/cureus.103629

**Published:** 2026-02-14

**Authors:** Yohei Otsuka, Toshinori Nishizawa, Toru Morikawa

**Affiliations:** 1 Department of General Medicine, Aso Iizuka Hospital, Iizuka, JPN; 2 Department of General Medicine, St. Luke's International Hospital, Tokyo, JPN; 3 Department of Data Science, Hyogo Medical University, Nishinomiya, JPN; 4 Department of General Medicine, Nara City Hospital, Nara City, JPN

**Keywords:** ct imaging necrosis, hemophagocytic syndrome, lymphoma differential diagnosis, tuberculosis, tuberculous lymphadenitis

## Abstract

A 78-year-old man presented with prolonged fever, weight loss, erythema, and necrotic lymphadenopathy. Initial suspicion of malignant lymphoma-associated hemophagocytic syndrome (HPS) arose from high Fluorodeoxyglucose (FDG) uptake in lymph nodes on PET/CT. However, lymph node biopsy revealed caseous necrosis and granulomas containing acid-fast bacilli, confirming tuberculosis-associated HPS. Diagnosis was delayed due to a negative interferon-gamma release assay and imaging and clinical features resembling malignancy. This case demonstrates that tuberculosis can act as a great mimicker, often presenting similarly to malignancy and complicating diagnosis. Necrotic lymphadenopathy with peripheral enhancement on CT and overlapping Maximum Standardized Uptake Value (SUV_max_) values on PET/CT should raise suspicion for tuberculosis in similar cases. Early lymph node biopsy is essential for differentiating tuberculosis from malignancy, ensuring appropriate treatment. Although anti-tuberculosis therapy combined with corticosteroids improved systemic inflammation, the patient’s frailty worsened. This case highlights the importance of considering tuberculosis in the differential diagnosis of systemic inflammation with cytopenia and lymphadenopathy to improve outcomes in severe conditions.

## Introduction

Tuberculosis, caused by *Mycobacterium tuberculosis*, remains a significant public health concern due to its high mortality rate [1]. It disproportionately affects populations with lower socioeconomic status, highlighting the deep intertwining of tuberculosis with psychosocial determinants [2]. Pulmonary tuberculosis typically presents with fever, night sweats, weight loss, cough, increased sputum production, and hemoptysis [3]. In contrast, extrapulmonary tuberculosis presents with organ-specific findings, which complicate diagnosis [4]. Tuberculous lymphadenitis, a form of extrapulmonary tuberculosis, is known to be difficult to distinguish from other causes of lymphadenopathy, such as sarcoidosis [5].

Tuberculous lymphadenitis can also lead to hemophagocytic syndrome (HPS), a life-threatening condition characterized by the excessive activation of macrophages and lymphocytes, which results in systemic inflammation [6]. Secondary HPS most frequently arises in the setting of malignancies, infections, and autoimmune diseases, yet tuberculosis-related cases are rare [7]. Diagnosis is often delayed, which increases the risk of mortality. Tuberculosis complicated by HPS carries a high mortality rate, indicating the need for early diagnosis and treatment [6].

Distinguishing HPS caused by tuberculous lymphadenitis from HPS associated with malignant lymphoma is crucial for timely and appropriate therapy. To our knowledge, literature specifically focusing on the diagnostic pitfalls between these two entities remains limited. Here, we present a case of HPS resulting from tuberculous lymphadenitis, illustrating the diagnostic challenge of differentiating it from HPS associated with lymphoma.

## Case presentation

A 78-year-old man with a history of hypertension presented to our hospital with a fever of unknown origin. The patient had no known history of exposure to individuals with active tuberculosis. He was born in Ikuno Ward, Osaka City, and relocated to Nara City, Nara Prefecture, 35 years ago. Notably, both Ikuno Ward and Nara Prefecture are recognized for having tuberculosis prevalence rates that exceed the national average. Although Japan is generally classified as a low-prevalence country, Osaka City remains a high-burden region. The patient was employed in the interior construction industry until the age of 70, during which time he participated in annual health screenings. Following his retirement, his medical surveillance was managed by a primary care physician via routine blood chemistry. 

 Four months prior to admission, the patient developed a persistent low-grade fever (37°C range) accompanied by the emergence of erythematous lesions on the extremities. Within one month, these lesions rapidly enlarged and disseminated to the trunk and neck. Despite the progressive nature of his illness, he initially reported no cough, fatigue, or night sweats.

Three months prior to admission, he was evaluated in a dermatology outpatient clinic. While the clinical presentation was suggestive of Sweet syndrome, a skin biopsy demonstrated only non-specific inflammatory cell infiltration, lacking the pathognomonic features required for a definitive diagnosis. Two months prior to admission, the patient was initiated on 15 mg/day of oral prednisolone for suspected idiopathic erythema, which resulted in the temporary resolution of both the fever and cutaneous symptoms. However, the low-grade fever recurred during a subsequent specialist-led taper, which reduced the dose to 10 mg/day after one week. Despite the recurrence of symptoms, the dose was further reduced to 5 mg/day, which led to a significant worsening of the erythema and fever (reaching 38°C).

 Seven days prior to admission, a rheumatology consultation was requested by the dermatologist. Clinical evaluation at that time identified right conjunctival injection, right-sided hearing loss, and left testicular tenderness. Given this multisystem involvement, systemic vasculitis, including Cogan syndrome, was suspected. At the time, a laboratory investigation revealed a significant systemic inflammatory response, with a C-reactive protein (CRP) level of 13.3 mg/dL, despite a white blood cell count within the normal range (4,300/µL). Serological testing revealed only rheumatoid factor positivity, while anti-neutrophil cytoplasmic antibodies (ANCA) were negative. Following an 18F-fluorodeoxyglucose positron emission tomography/computed tomography (FDG-PET/CT) scan, the patient was referred to the department of general internal medicine for an evaluation of suspected systemic vasculitis or malignant lymphoma. Upon admission, the patient’s systemic condition had markedly deteriorated, characterized by persistent fever and anorexia; notably, a cumulative weight loss of 9 kg was documented over the four-month course of his illness.

**Figure 1:**
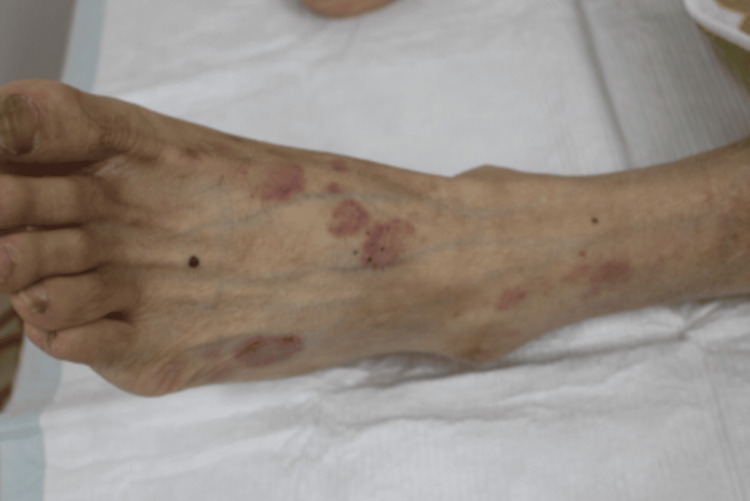
Erythematous lesions on the left foot.

At the time of admission, physical examination revealed a febrile state with a temperature of 38.1°C. Other vital signs remained stable: blood pressure, 104/69 mmHg; pulse, 74 beats/min; respiratory rate, 20 breaths/min; and oxygen saturation, 96% on room air. On physical examination, a discrete, 1 cm, painless lymph node with a rubbery consistency was palpable in the right supraclavicular fossa. In addition, several painless erythematous lesions measuring approximately 2 cm in diameter, without palpable induration, were observed on the left dorsum of the foot (Figure 1). Heart sounds were normal, breath sounds were clear, and no lower extremity edema was observed. No conjunctival hyperemia, hepatosplenomegaly, or testicular tenderness was present.

Hematologic evaluation revealed pancytopenia (white blood cell count, 2,770/μL; hemoglobin, 9.5 g/dL; platelet count, 123,000/μL) with rouleaux formation on the peripheral smear; blast cells were absent. Biochemical profiling indicated a profound hyperinflammatory state, characterized by marked elevations in ferritin (5,975 ng/mL), soluble interleukin-2 receptor (1,742 U/mL), C-reactive protein (13.49 mg/dL), and lactate dehydrogenase (422 U/L). Fibrinogen was elevated at 679 mg/dL, while triglyceride levels were 109 mg/dL (Table 1). Tests for antinuclear antibodies, ANCA, syphilis, Human immunodeficiency virus (HIV), and the interferon-gamma release assay (IGRA) for tuberculosis were negative.

**Table 1:** Laboratory findings on admission.

Variable	Result	Unit	Reference range
Complete blood count			
White blood cell count	2,770	/μL	3,300-8,600
Neutrophils	66.7	%	37-72
Lymphocytes	21.7	%	20-50
Monocytes	11.2	%	0-14
Eosinophils	0	%	0-6
Hemoglobin	9.5	g/dL	13.7-16.8
Mean corpuscular volume (MCV)	96.0	fL	83.6-98.2
Platelet count	123,000	/μL	158,000-348,000
Coagulation			
PT-INR (prothrombin time-international normalized ratio)	1.22		0.9-1.2
APTT (activated partial thromboplastin time)	35.3	sec	24-34
Fibrinogen	679	mg/dL	180-380
D-dimer	3.8	μg/mL	< 1.0
Biochemistry			
Total protein	6.6	g/dL	6.6-8.1
Albumin	2.8	g/dL	4.1-5.1
Aspartate aminotransferase (AST)	46	U/L	13-30
Alanine aminotransferase (ALT)	30	U/L	10-42
Lactate dehydrogenase (LDH)	422	U/L	124-222
Alkaline phosphatase (ALP)	99	U/L	38-113
Gamma-glutamyl transpeptidase (γ-GTP)	32	U/L	13-64
Total bilirubin	0.60	mg/dL	0.4-1.5
Creatine kinase (CK)	33	U/L	59-248
Blood urea nitrogen	21.0	mg/dL	8-20
Creatinine	1.06	mg/dL	0.65-1.07
Triglycerides	109	mg/dL	30-149
Sodium (Na)	132	mmol/L	138-145
Potassium (K)	4.2	mmol/L	3.6-4.8
Chloride (Cl)	98	mmol/L	101-108
Inflammation and Immunology			
C-reactive protein (CRP)	13.49	mg/dL	<0.14
Ferritin	5,975	ng/mL	50-200
Erythrocyte sedimentation rate (ESR)			
1 hour	99	mm	<10
2 hours	105	mm	<25
Rheumatoid factor (RF)	74	IU/mL	<15
Soluble interleukin-2 receptor (sIL-2R)	1,742	U/mL	122-496

**Figure 2:**
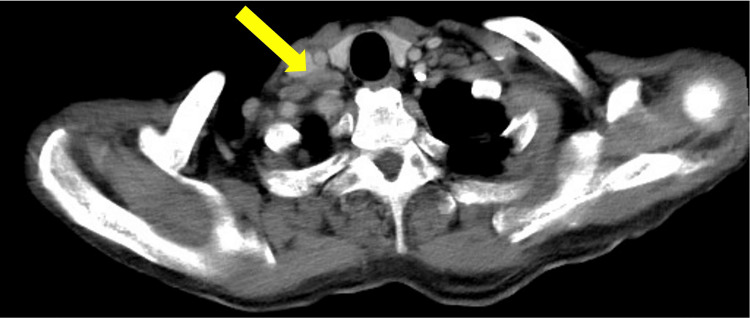
Contrast-enhanced CT of the chest and pelvis showing infraclavicular lymphadenopathy. CT revealed right supraclavicular lymphadenopathy (arrow) with peripheral heterogeneous enhancement and central low attenuation.

Contrast-enhanced computed tomography (CT) revealed multiple enlarged lymph nodes in the right supraclavicular fossa and the mediastinum, with peripheral heterogeneous enhancement and central low-density areas suggestive of necrosis (Figure 2). Mild splenomegaly was observed, whereas hepatomegaly and other organ abnormalities were not present. The radiology report at our institution suggested malignant lymphoma.

**Figure 3:**
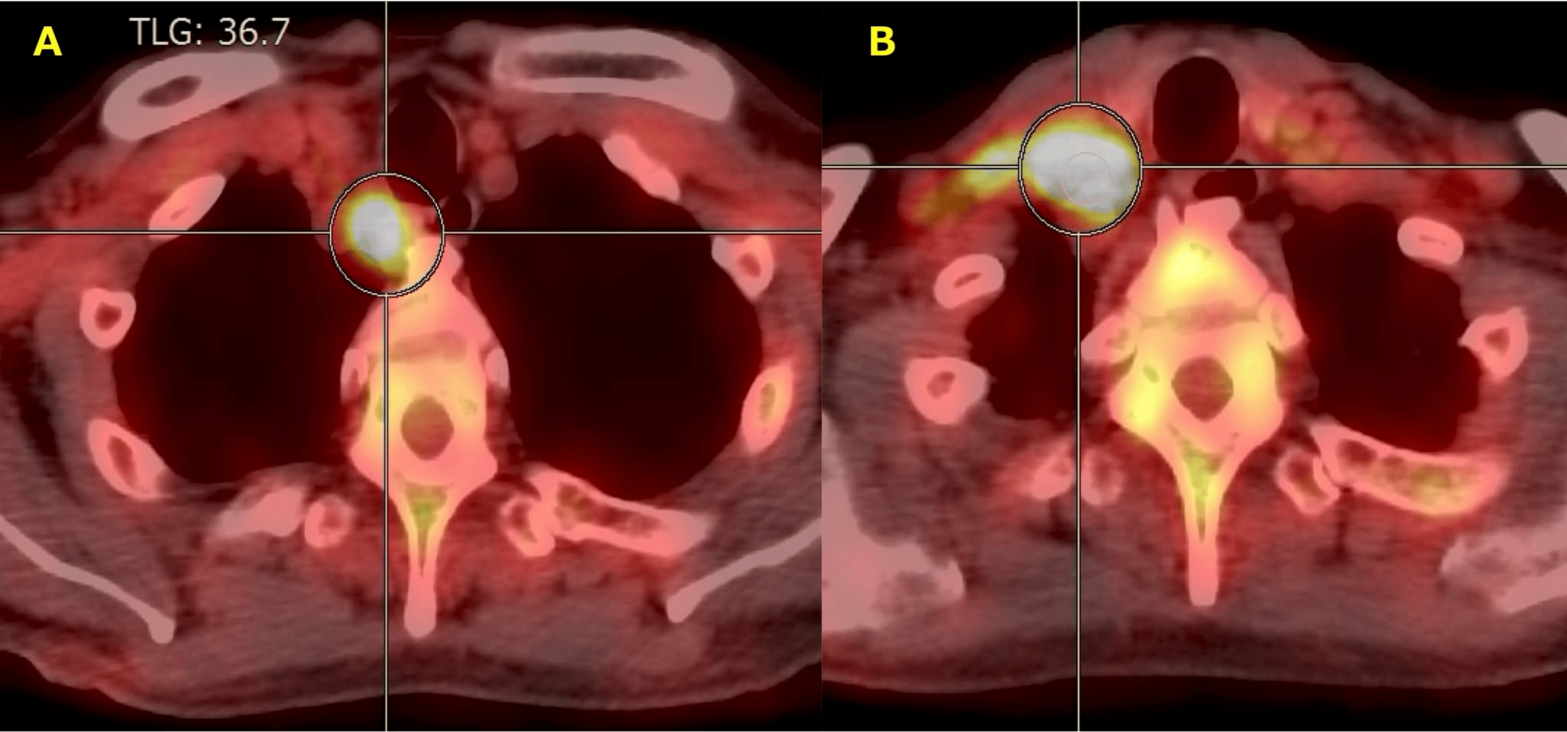
FDG-PET/CT findings of lymphadenopathy. FDG-PET/CT demonstrated multiple hypermetabolic enlarged lymph nodes (SUV_max _= 8.5-14.9) involving the right upper mediastinum (A) and right supraclavicular fossa (B).

FDG-PET/CT demonstrated multiple enlarged right supraclavicular lymph nodes with high FDG uptake (maximum standardized uptake value (SUV_max _= 8.5-14.9), as well as diffuse FDG uptake in the bone marrow, particularly in the sternum and bilateral femurs (Figure 3).

We initially suspected malignant lymphoma based on progressive lymphadenopathy, weight loss, fever, skin rash, elevated lactate dehydrogenase levels, findings on contrast-enhanced CT and the radiologist’s report, and diffuse FDG uptake in the bone marrow on FDG-PET/CT. At that time, lymphadenopathy was the predominant clinical feature, and there was no relevant exposure history; therefore, tuberculosis was not suspected. 

Bone marrow aspiration and trephine biopsy demonstrated hemophagocytosis within a normocellular to hypocellular marrow. There was no evidence suggestive of mycobacterial infection; acid-fast bacilli staining was negative, and histopathological examination revealed an absence of granulomatous inflammation. Hemophagocytic syndrome secondary to malignant lymphoma was suspected, and a right supraclavicular lymph node biopsy was scheduled. During hospitalization, he had a fever (37-39°C) and reduced food intake.

**Figure 4:**
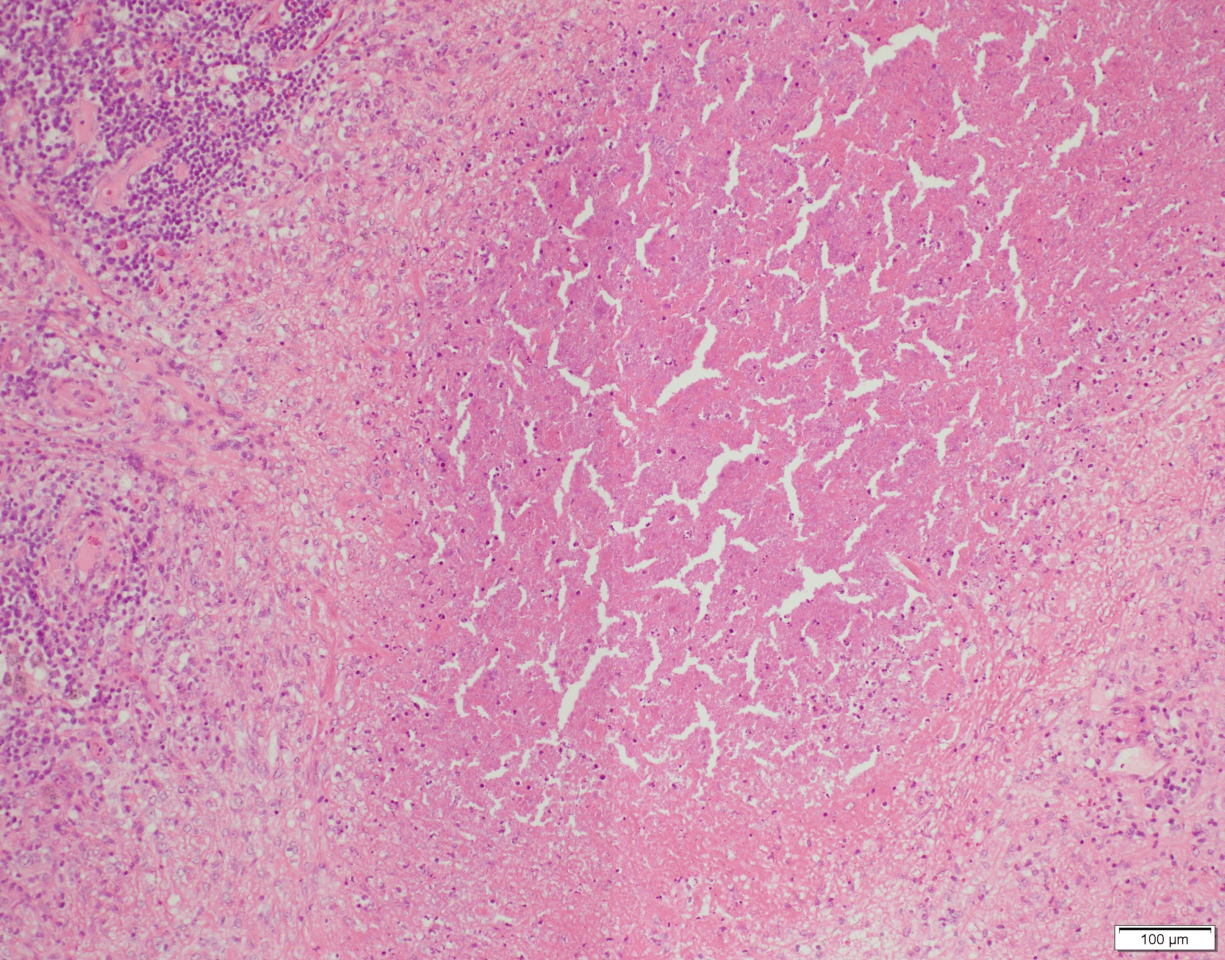
Hematoxylin and eosin staining of the lymph node revealed areas of caseous necrosis.

**Figure 5:**
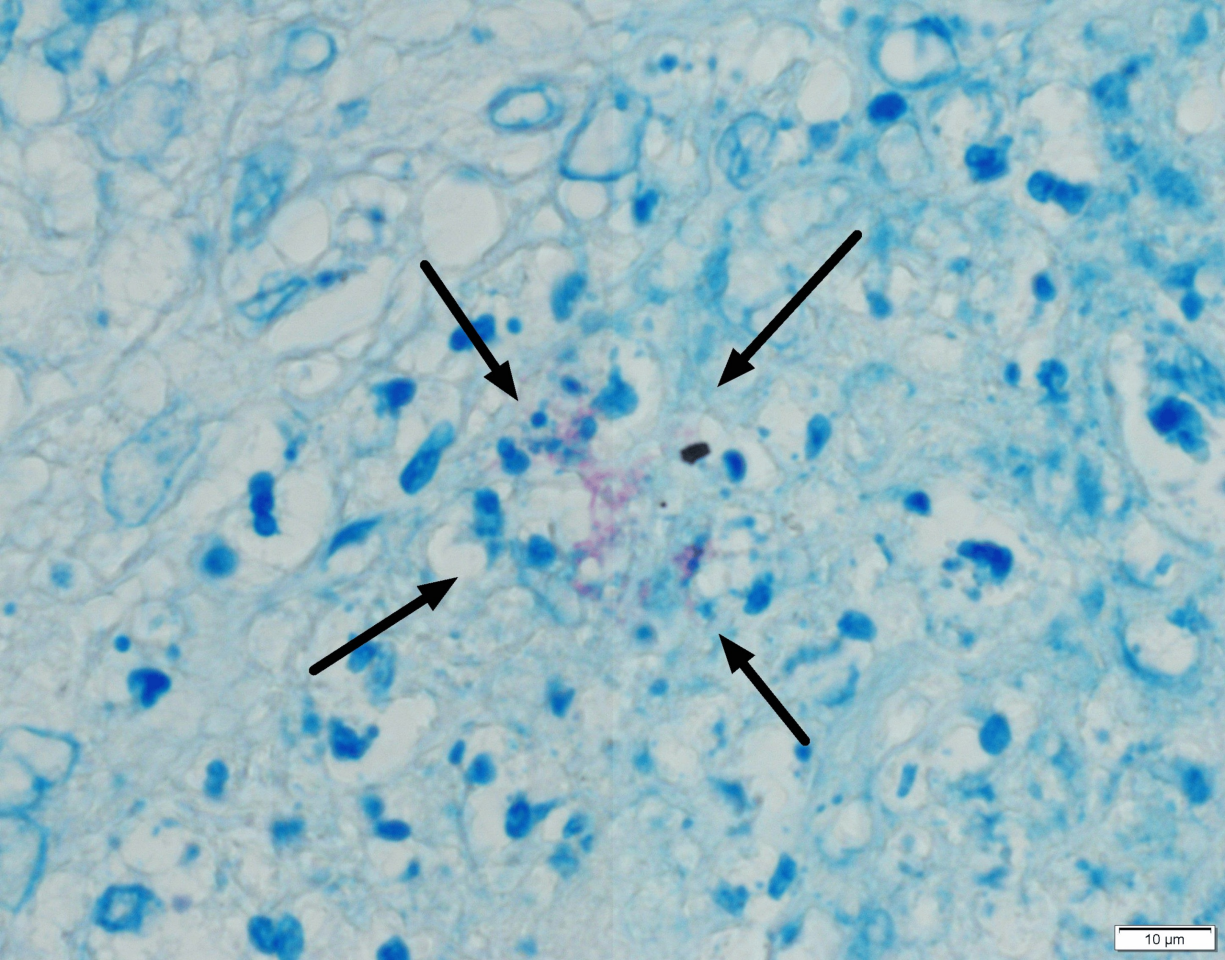
Mycobacterium tuberculosis demonstrated by Ziehl-Neelsen (acid-fast) staining of the lymph node.

On the eighth day of hospitalization, an excisional lymph node biopsy was performed. The histopathological examination revealed caseous necrosis and granulomatous inflammation, which is characterized by the presence of epithelioid cells and multinucleated giant cells (Figure 4). Ziehl-Neelsen staining confirmed the presence of acid-fast bacilli (Figure 5).

Since the initial differential diagnosis was malignant lymphoma, the surgical specimen was fixed in formalin, which rendered it unsuitable for conventional mycobacterial culture or molecular testing. To facilitate microbiological confirmation, residual fresh lymph node tissue originally allocated for flow cytometry was used. The Xpert *Mycobacterium tuberculosis*/Rifampicin resistance (MTB/RIF) assay and mycobacterial culture of this salvaged nodal tissue were both positive for *Mycobacterium tuberculosis*, confirming a diagnosis of tuberculous lymphadenitis. Furthermore, Xpert MTB/RIF assay and mycobacterial cultures of sputum, gastric fluid, stool, blood, urine, and bone marrow were all negative. 

He met four of the hemophagocytic lymphohistiocytosis-2004 (HLH-2004) criteria: fever, cytopenia in two cell lines, hemophagocytosis in the bone marrow, and elevated ferritin levels [8]. Although five criteria are required for a definitive HLH diagnosis, these findings were consistent with hemophagocytic syndrome secondary to tuberculosis. Consequently, a diagnosis of tuberculosis-associated HPS was made.

Due to the difficulty of providing tuberculosis treatment at our hospital and the patient's poor general condition caused by tuberculosis-associated hemophagocytic syndrome, he was transferred to a higher-level medical facility. Upon establishing the diagnosis, quadruple anti-tuberculosis therapy (isoniazid, rifampicin, ethambutol, and pyrazinamide) was initiated, combined with adjunctive prednisolone. Although the patient demonstrated rapid defervescence and significant improvement in CRP and LDH levels, his general frailty and functional decline progressed due to the prolonged consumptive nature of the disease. This physical deterioration led to severe dysphagia and difficulty with oral intake. Consequently, in view of the patient’s progressive decline and the shift in the goals of care toward end-of-life support, follow-up imaging was not performed. The patient was transferred to a long-term care facility to receive palliative care.

## Discussion

This case highlights the importance of taking a detailed residential history when diagnosing tuberculous lymphadenitis, especially in areas with a diverse disease burden. Although Japan achieved low-prevalence status in 2021, Osaka City's decline in tuberculosis incidence has lagged behind the national average [9]. Recent geodemographic analyses have identified distinct hotspots within the city where rates remain disproportionately high [9]. Our patient lived in a district with a high tuberculosis burden. The combination of this geographic risk factor and constitutional symptoms such as night sweats, fever, and weight loss should have raised suspicion for tuberculosis, placing it alongside malignant lymphoma in the initial differential diagnosis. Although the institutional radiology report primarily suggested malignant lymphoma and did not initially include tuberculosis in the differential diagnosis, this case highlights the critical importance of taking a comprehensive medical history. Incorporating psychosocial and geographical backgrounds is essential to avoid premature diagnostic closure, even when imaging findings, such as CT or PET/CT, favor an alternative diagnosis. 

In cases of lymphadenopathy with heterogeneous enhancement on contrast-enhanced CT, suggesting internal necrosis, the differential diagnosis includes both malignant lymphoma and tuberculous lymphadenitis. Active tuberculosis-related lymphadenitis often presents as poorly enhanced regions in lymph nodes, corresponding pathologically to caseous necrosis [10]. These findings are reliable indicators of disease activity. Additionally, contrast enhancement patterns differ between tuberculosis and lymphoma: peripheral enhancement is observed in 78% of tuberculosis cases, whereas homogeneous enhancement is found in 83% of non-Hodgkin’s and Hodgkin’s lymphomas. Mixed enhancement patterns are rare, occurring in approximately 10% of lymphoma cases [11]. In our case, contrast-enhanced CT revealed lymph nodes with marginal heterogeneous enhancement and internal low-attenuation areas, indicating the need for early consideration of tuberculosis in similar presentations.

Furthermore, although FDG uptake on PET/CT in lymph nodes frequently raises suspicion for malignant lymphoma, the mean FDG SUV_max_ for sarcoidosis, tuberculosis, Hodgkin’s lymphoma, and Non-Hodgkin’s lymphoma are 12.7, 13.4, 8.2, and 8.8, respectively [12]. These values suggest that SUV_max_ alone does not reliably differentiate malignant lymphoma from other causes of lymphadenopathy. Formalin fixation of lymph node specimens precludes mycobacterial culture; where tuberculosis is a consideration, fresh tissue for PCR and culture should be obtained whenever feasible. In cases of necrotic localized lymphadenopathy, particularly in tuberculosis-endemic regions and in elderly patients with cytopenia, it is essential to include tuberculous lymphadenitis and HPS in the differential diagnosis and to proceed with lymph node biopsy.

When malignant lymphoma-associated HPS is suspected, tuberculosis-associated HPS should also be considered for evaluation. Common causes of HPS include malignancy, infection, and autoimmune disease. While tuberculosis-associated HPS is rare, lymphoma-associated HPS occurs approximately ten times more frequently [7]. In tuberculosis-associated HPS, early diagnosis and treatment are essential, as combination therapy with antitubercular drugs and steroids has been shown to reduce mortality. However, the overall mortality rate remains high, at approximately 45%, emphasizing the importance of timely intervention [6]. Reports indicate that Mycobacterium tuberculosis can be detected in bone marrow in 78.4% of cases with tuberculosis-associated HPS [13]. Thus, in cases where lymph node biopsy is delayed or tuberculosis-associated HPS is suspected, performing tuberculosis PCR on bone marrow may allow earlier diagnosis. In our case, although *Mycobacterium tuberculosis* was not detected in the bone marrow, findings suggesting tuberculosis, including erythema, fever, and weight loss, were present.

However, the negative interferon-gamma release assay (IGRA) result contributed to the diagnostic delay. It is important to note that IGRA is primarily a screening tool for latent tuberculosis infection and cannot be used to definitively rule out active tuberculosis. Notably, in tuberculosis-associated HPS, only 10.5% of patients show positive results on tuberculin skin testing or IGRA, indicating the limited reliability of these tests for ruling out tuberculosis-associated HPS [13]. Given that the patient had cytopenia and was being treated with corticosteroids, which may suppress immune responses, the IGRA result was considered to be less reliable.

Unexplained erythema accompanying fever and weight loss should also prompt consideration of tuberculosis. Cutaneous manifestations like erythema are non-specific. However, a skin biopsy supplemented by mycobacterial culture and molecular testing may facilitate a more definitive diagnosis [14]. Tuberculosis is commonly associated with erythema nodosum, particularly in endemic regions where a strong correlation exists. Studies report that approximately 6% of erythema nodosum cases and 21% of infection-related erythema nodosum cases are linked to tuberculosis [15]. Although rare, tuberculosis has also been associated with Sweet’s syndrome, or acute febrile neutrophilic dermatosis. Sweet’s syndrome is characterized by a dysregulated immune response, often associated with a T helper type 1 (Th1)-dominant cytokine profile [16]. In this case, erythema resembling Sweet’s syndrome was noted three months prior to admission. This finding highlighted the importance of considering tuberculosis-associated dermatoses in patients with unexplained erythema and fever.

## Conclusions

In conclusion, this case of tuberculosis-associated HPS was challenging to distinguish from malignant lymphoma-associated HPS. Tuberculosis, often referred to as the great mimicker, can present with nonspecific symptoms that complicate diagnosis and increase the risk of errors. It is essential to maintain a high index of suspicion for tuberculosis in the differential diagnosis of unexplained lymphadenopathy, fever, cytopenia, and erythema, particularly in patients with a history of residence in tuberculosis-endemic areas. Early recognition and diagnosis are crucial for initiating appropriate treatment and improving patient outcomes.

## References

[1] Yang J, Zhang L, Qiao W, Luo Y (2023). Mycobacterium tuberculosis: pathogenesis and therapeutic targets. MedComm (2020).

[2] Natarajan A, Beena PM, Devnikar AV, Mali S (2020). A systemic review on tuberculosis. Indian J Tuberc.

[3] Lawn SD, Zumla AI (2011). Tuberculosis. Lancet.

[4] Jawed A, Tharwani ZH, Siddiqui A (2023). Better understanding extrapulmonary tuberculosis: a scoping review of public health impact in Pakistan, Afghanistan, India, and Bangladesh. Health Sci Rep.

[5] Hoornaert E, Yildiz H, Pothen L (2024). A comparison study of lymph node tuberculosis and sarcoidosis involvement to facilitate differential diagnosis and to establish a predictive score for tuberculosis. Pathogens.

[6] Fauchald T, Blomberg B, Reikvam H (2023). Tuberculosis-associated hemophagocytic lymphohistiocytosis: a review of current literature. J Clin Med.

[7] Ramos-Casals M, Brito-Zerón P, López-Guillermo A, Khamashta MA, Bosch X (2014). Adult haemophagocytic syndrome. The Lancet.

[8] Henter JI, Horne A, Aricó M (2007). HLH-2004: diagnostic and therapeutic guidelines for hemophagocytic lymphohistiocytosis. Pediatr Blood Cancer.

[9] Yamamoto K, Takeuchi S, Nakaya T (2025). Geodemographic analysis of socioeconomic area disparities in tuberculosis incidence in Osaka City, Japan. Sci Rep.

[10] Moon WK, Im JG, Yeon KM, Han MC (1998). Mediastinal tuberculous lymphadenitis: CT findings of active and inactive disease. AJR Am J Roentgenol.

[11] Tang SS, Yang ZG, Deng W, Shao H, Chen J, Wen LY (2012). Differentiation between tuberculosis and lymphoma in mediastinal lymph nodes: Evaluation with contrast-enhanced MDCT. Clin Radiol.

[12] Rayamajhi SJ, Mittal BR, Maturu VN (2016). (18)F-FDG and (18)F-FLT PET/CT imaging in the characterization of mediastinal lymph nodes. Ann Nucl Med.

[13] Kurver L, Seers T, van Dorp S, van Crevel R, Pollara G, van Laarhoven A (2024). Tuberculosis-associated hemophagocytic lymphohistiocytosis: diagnostic challenges and determinants of outcome. Open Forum Infect Dis.

[14] Gramminger C, Biedermann T (2025). Recognising cutaneous tuberculosis. J Dtsch Dermatol Ges.

[15] Laborada J, Cohen PR (2021). Tuberculosis-associated erythema nodosum. Cureus.

[16] Joshi TP, Friske SK, Hsiou DA, Duvic M (2022). New practical aspects of sweet syndrome. Am J Clin Dermatol.

